# Melatonin Pretreatment Confers Heat Tolerance and Repression of Heat-Induced Senescence in Tomato Through the Modulation of ABA- and GA-Mediated Pathways

**DOI:** 10.3389/fpls.2021.650955

**Published:** 2021-03-25

**Authors:** Mohammad Shah Jahan, Sheng Shu, Yu Wang, Md. Mahadi Hasan, Ahmed Abou El-Yazied, Nadiyah M. Alabdallah, Dina Hajjar, Muhammad Ahsan Altaf, Jin Sun, Shirong Guo

**Affiliations:** ^1^Key Laboratory of Southern Vegetable Crop Genetic Improvement in Ministry of Agriculture, College of Horticulture, Nanjing Agricultural University, Nanjing, China; ^2^Department of Horticulture, Faculty of Agriculture, Sher-e-Bangla Agricultural University, Dhaka, Bangladesh; ^3^State Key Laboratory of Grassland Agro-Ecosystems, School of Life Sciences, Lanzhou University, Lanzhou, China; ^4^Department of Horticulture, Faculty of Agriculture, Ain Shams University, Cairo, Egypt; ^5^Department of Biology, College of Science, Imam Abdulrahman Bin Faisal University, Dammam, Saudi Arabia; ^6^Department of Biochemistry, College of Science, University of Jeddah, Jeddah, Saudi Arabia; ^7^Center for Terrestrial Biodiversity of the South China Sea, School of Life and Pharmaceutical Sciences, Hainan University, Haikou, China

**Keywords:** leaf senescence, chlorophyll degradation, high temperature, melatonin, tomato

## Abstract

Heat stress and abscisic acid (ABA) induce leaf senescence, whereas melatonin (MT) and gibberellins (GA) play critical roles in inhibiting leaf senescence. Recent research findings confirm that plant tolerance to diverse stresses is closely associated with foliage lifespan. However, the molecular mechanism underlying the signaling interaction of MT with GA and ABA regarding heat-induced leaf senescence largely remains undetermined. Herein, we investigated putative functions of melatonin in suppressing heat-induced leaf senescence in tomato and how ABA and GA coordinate with each other in the presence of MT. Tomato seedlings were pretreated with 100 μM MT or water and exposed to high temperature (38/28°C) for 5 days (d). Heat stress significantly accelerated senescence, damage to the photosystem and upregulation of reactive oxygen species (ROS), generating *RBOH* gene expression. Melatonin treatment markedly attenuated heat-induced leaf senescence, as reflected by reduced leaf yellowing, an increased Fv/Fm ratio, and reduced ROS production. The *Rbohs* gene, chlorophyll catabolic genes, and senescence-associated gene expression levels were significantly suppressed by MT addition. Exogenous application of MT elevated the endogenous MT and GA contents but reduced the ABA content in high-temperature-exposed plants. However, the GA and ABA contents were inhibited by paclobutrazol (PCB, a GA biosynthesis inhibitor) and sodium tungstate (ST, an ABA biosynthesis inhibitor) treatment. MT-induced heat tolerance was compromised in both inhibitor-treated plants. The transcript abundance of ABA biosynthesis and signaling genes was repressed; however, the biosynthesis genes MT and GA were upregulated in MT-treated plants. Moreover, GA signaling suppressor and catabolic gene expression was inhibited, while ABA catabolic gene expression was upregulated by MT application. Taken together, MT-mediated suppression of heat-induced leaf senescence has collaborated with the activation of MT and GA biosynthesis and inhibition of ABA biosynthesis pathways in tomato.

## Introduction

Recently, high temperature has become a great threat to sessile plants; it is characterized by hastening leaf senescence (Jespersen et al., 2016) and leading to a remarkable decline in plant growth (Soltani et al., 2019). It is projected that global temperatures will increase from 1.8 to 4.0°C by 2100 (Parry and Pizer, 2007). Leaf senescence is a fine-tuned mechanism that is intensely complicated by diverse intrinsic factors, such as cell death (Ghanem et al., 2012), phytohormones (Zhang and Guo, 2018), senescence-associated genes (Li et al., 2017; Xiao et al., 2017), transcription factors (Ma et al., 2018b), and environmental factors like darkness (Weaver et al., 1998), detachment (He and Gan, 2002), drought (Lee et al., 2012), salinity or alkalinity (Yang et al., 2011; Xiao et al., 2015), and high temperature (Xu and Huang, 2007; Zheng et al., 2016). The decline in Chl content is the most prominent feature of natural or stress-induced leaf senescence (Hörtensteiner, 2006), which is important for the absorption of light and the redistribution of excitation energy in the photosynthetic electron transport chain (Grossman et al., 1995). Senescence-associated gene (SAG) expression is upregulated during the onset of senescence, while the transcripts of photosynthesis-related genes are decreased (Hörtensteiner, 2006). Leaf yellowing is manifested in senescent leaves due to the negative functioning of chlorophyll catabolic enzymes, particularly Chl a reductase (HCAR), pheophytin pheophorbide hydrolyase (PPH), non-yellow coloring 1 (NYC1), NYC1-like (NOL), and pheide a oxidase (PAO) (Barry, 2009; Hörtensteiner, 2009). The transcription of Chl catabolic genes (CCGs) is directly associated with the severity of normal or stress-induced leaf senescence in many plant species (Schelbert et al., 2009; Sakuraba et al., 2012; Zhang et al., 2016b). Another essential characteristic of leaf senescence is overaccumulation of ROS (Wu et al., 2012; Gütle et al., 2016). ROS homeostasis and the redox state regulate growth- or senescence-associated cell death. In plants, ROS are generally produced by many enzymes (Apel and Hirt, 2004). Respiratory burst oxidase homologs (Rbohs) are extensively studied ROS-creating enzymes in plants (Sagi and Fluhr, 2006; Suzuki et al., 2011). Most research findings have highlighted that Rbohs are implicated in diverse distinct signaling networks and acclimation to various stresses (Suzuki et al., 2011; Marino et al., 2012; Kaur et al., 2014).

Several phytohormones, including ABA, jasmonic acid, ethylene, and salicylic acid, promote leaf senescence; while leaf senescence is restricted by GA, auxins, cytokinins, and polyamines (Jibran et al., 2013; Kim et al., 2016; Woo et al., 2019). ABA content and ABA biosynthesis and signaling gene expression are enhanced in the course of leaf senescence (Liang et al., 2014; Mao et al., 2017). The 9-cis-epoxycarotenoid dioxynease (NCED) is the key regulatory enzyme and is considered a rate-limiting step for ABA biosynthesis (Nambara and Marion-Poll, 2005). Chl catabolic gene expression is also regulated in the presence of AREB/ABF members in Arabidopsis (Gao et al., 2016). A large number of gibberellins are found in the plant kingdom but a limited version of GAs is proactive and helpful for plant development (Yamaguchi et al., 1998). Beyond other activities, GAs are used to prolong leaf senescence (Beevers, 1966; Whyte and Luckwill, 1966; Lü et al., 2014; Xiao et al., 2019).

ABA and GA participate in diverse as well as antagonistic roles in plant development processes, flowering, and regulate various environmental stimuli from the physiological to the molecular level (Weiss and Ori, 2007; Golldack et al., 2013; Liu and Hou, 2018). Heat treatment decreases GA and increases ABA content in Arabidopsis during seed germination (Toh et al., 2008). The increased ABA content in germinating seeds during heat stress causes upregulation of ABA biosynthesis genes; by contrast, a lower GA content in imbibed seeds leads to the downregulation of GA biosynthesis gene expression (Toh et al., 2008). The key seed development dimer FUS3 and ABA metabolic genes are activated during seed germination, whereas GA catabolic gene expression is restricted under heat stress, leading to delayed germination (Chiu et al., 2012). Correspondingly, the DELLA proteins RGA or GAI, as well as *ABI3* and *ABI5*, distinctly induce small ubiquitin-related modifiers (SOMs) that modulate GA and ABA biosynthesized genes under heat stress in Arabidopsis (Lim et al., 2013).

Melatonin acts as an essential antioxidant that leads to prolonged leaf senescence under stress environments (Arnao and Hernández-Ruiz, 2015). Exogenous application of melatonin on tryptophan decarboxylase (*TDC*), serotonin N-acetyltransferase (*SNAT*), tryptamine 5-hydroxylase (*T5H*) and caffeic acid O-methyltransferase (*COMT*) transgenic plants (Byeon et al., 2015) enhanced melatonin content (Zhang N. et al., 2014), which inhibit chlorophyll reduction and downregulation of CCE and SAG gene expression under diverse stresses (Wang et al., 2012; Liang et al., 2015; Shi et al., 2015b; Ma et al., 2018a). In addition, melatonin is a well-known ROS scavenger and excellent antioxidant that scavenges excess ROS (Li et al., 2012; Ahammed et al., 2018; Jahan et al., 2020) and inhibits the stress-induced senescence mechanism in plants. Melatonin efficacy in terms of inhibition of senescence-induced damage has been reported in some previous studies, including Arabidopsis, kiwi, grapes, rice, barley, Chinese flowering cabbage and ryegrass (Arnao and Hernández-Ruiz, 2009; Wang et al., 2013; Liang et al., 2015; Zhang et al., 2016a; Liang et al., 2018; Shi et al., 2019; Tan et al., 2019). Melatonin inhibits senescence-related gene expression during drought-induced leaf senescence in apple trees (Wang et al., 2013). A recent experiment showed that melatonin prolongs senescence in kiwifruit leaves via enhancement of the antioxidant defense system and upregulation of flavonoid biosynthesis (Liang et al., 2018). In addition, melatonin is involved in eliminating Chl degradation by suppressing Chl degradation enzymes (Weeda et al., 2014). Melatonin application led to enhanced drought stress-induced leaf senescence, resulting in decreased ABA production and ABA biosynthesis gene expression (Li et al., 2015). Interestingly, melatonin treatment in Chinese flowering cabbage prolonged storage-induced leaf senescence through restricted ABA production and lowered Chl reduction associated with ABA signaling transcription factors, i.e., *BrABF1*, *BrABF4* and *BrABI5* (Tan et al., 2019). Arnao and Hernández-Ruiz (2009) showed that both melatonin and cytokinin treatment effectively reduced dark-induced Chl loss in barley leaves, and the effects were more pronounced than those of cytokinin treatment alone. The inherent ability of melatonin could help to mitigate diverse stresses through linking with other phytohormones (Arnao and Hernández-Ruiz, 2014). Despite ample documentation of the roles of melatonin in terms of stress tolerance mechanism, melatonin-mediated heat-induced leaf senescence with other hormones is still not fully understood, and it is unclear how melatonin interacts with GA and/or ABA signaling networks to mitigate senescence. In the present experiment, we demonstrated that melatonin functioned synergistically with GA while acting antagonistically with ABA in their biosynthesis and signaling pathways to prolong heat-induced leaf senescence in tomato.

## Materials and Methods

### Planting Materials and Growing Conditions

Tomato (*Solanum lycopersicum* Cv. Hezuo 903) seeds were used as the test material for this experiment. Sterilized seeds were incubated for germination on moistened filter papers in a dark place at 28 ± 1°C for 30 h. After germination, seeds were placed in plastic trays filled with organic substrates (peat and vermiculite: 2:1, v:v) in an artificial climate growth chamber. The following growth environmental conditions were maintained: temperature: 28/19 ± 1°C (day/night), relative humidity: 65–75%, and 12 h photoperiods (PAR 300 μmol m^–2^ s^–1^). When the second leaves were fully expanded, seedlings were shifted into the same growth substrate mixtures, and every alternate day, they were irrigated with nutrient solution.

### Treatment Application and Sample Collection

When the seedlings attained the fourth leaf stage, half of the seedlings were foliar sprayed with melatonin at a concentration of 100 μM every 2 days and continued for seven (7) days, while the other half of the seedlings were hydrosprayed with distilled water. One week after treatments, melatonin and water-treated seedlings were subjected to high-temperature stress at 38/28°C (16/8 h) for 5 days. Leaves sampled (third leaf from the top to bottom) were collected at different time points for further biochemical analysis.

We applied ABA and GA inhibitors to verify the function of GA and ABA in MT-mediated heat tolerance. One week after foliar spraying with melatonin or water in the abovementioned volume, seedlings underwent different inhibitor treatments. The plants were foliar sprayed with 1 mM paclobutrazol (PCB, a GA biosynthesis inhibitor) and 1 mM sodium tungstate (ST, an ABA biosynthesis inhibitor) before 12 h of heat stress at 38/28°C (16/8 h) for 24 h, after which leaf samples were collected for endogenous GA and ABA measurement.

### Evaluation of Leaf Senescence

Plant physiological attributes, including chlorophyll fluorescence, gas exchange parameters, chlorophyll content, relative electrolytic leakage (REL), malondialdehyde (MDA), and hydrogen peroxide (H_2_O_2_), were applied for the assessment of leaf senescence. Approximately 0.50 g of composite leaf tissue was extracted in 80% cold acetone to determine the chlorophyll contents and the extraction was centrifuged to collect the supernatant, and the chlorophyll content was determined spectrophotometrically (Arnon, 1949). Two essential fluorescence attributes net photosynthetic rate (*P*_*n*_) and stomatal conductance (*G*_*s*_), were measured with a portable photosynthesis system (Li-6400; LI-COR, Inc., Lincoln, NE, United States) from 10.00 am to 11.00 am. The cuvette conditions were maintained as follows: 25°C temperature, 70% relative humidity, 800 μmol photons m^–2^s^–1^ PPFD (photosynthetic photon flux density), and 380 ± 10 μmol mol^–1^ external CO_2_ concentration (Ahammed et al., 2020a; Hasan et al., 2020).

The maximum PSII quantum yield (Fv/Fm) was monitored as described by Maxwell and Johnson (2000), and an IMAGING-PAM chlorophyll fluorescence analyzer (Heinz Walz, Effeltrich, Germany) was used Fv/Fm measurement. Images were taken using a charge-coupled device (CCD) at the emitted fluorescence.

According to Khan et al. (2017) and Jahan et al. (2019b), we calculated the relative electrolyte leakage of the stressed leaves, and the REL was estimated using the following formula:

$$\mathrm{REL}\left(\%\right)=\frac{\mathrm{EC1}-\mathrm{EC0}}{\mathrm{EC2}-\mathrm{EC0}}\times 100$$

The MDA (malondialdehyde) content was determined following the instructions of Heath and Packer (1968). The concentration of H_2_O_2_ in stressed tomato leaves was measured according to Velikova et al. (2000) instructions.

### Determination of Melatonin Content

The melatonin content of tomato leaves was extracted using a commercial melatonin ELISA Kit (Qingdao Sci-tech Innovation Quality Testing Co., Ltd., Qingdao, China) following the company’s instructions. Briefly, 0.10 g of composite leaf sample was homogenized in 150 μL of 1 × stabilizer and 750 μL of ethyl acetate followed by proper vortexing. The homogenate was then extracted and evaporated to dryness, and the pellet was dissolved in a stabilizer solution. For the enzyme-linked immunosorbent assay, 100 μL of melatonin extract and 50 μL of 1 × melatonin antibody were kept in the microplate and incubated at 25°C on a plate shaker at 500 rpm for 1 h. Melatonin content was assessed by a microplate reader (Pow-erWaveX, Bio-Tek, United States), and on the basis of the standard curve, the concentration was computed using the reading of the absorbance at 450 nm.

### Quantification of Endogenous ABA Content

Approximately 500 mg of composite fresh tomato leaves was granulated in liquid nitrogen and then blended in ice-cold 80% methanol (v/v) extraction solution. The extracts were centrifuged at 12,000 g for 15 min at 4°C. The whole supernatant was run through a Sep-Pak C18 cartridge (Waters, Milford, MA, United States) to reduce the extraneous materials. According to the manufacturer’s protocols, endogenous ABA was estimated with an ABA ELISA Kit (Qingdao Sci-tech Innovation Quality Testing Co., Ltd., Qingdao, China).

### Analysis of Endogenous GA Content

Approximately 0.50 g of fresh composite tomato leaf sample was blended in 10 mL of ice-cold 80% methanol (v/v) extraction solution, including 1 mM butylated hydroxytoluene. The extraction solution was incubated at 4°C for 4 h, and the supernatant was transferred to a 10 mL centrifuge tube. Afterward, the supernatant was centrifuged for 8 min at 3,500 g. After incubation for 4 h at 4°C, the mixture was transferred to a 10 mL centrifuge tube and then centrifuged at 3,500 g for 8 min. The whole supernatant was run through a Sep-Pak C18 cartridge (Waters, Milford, MA, United States) to reduce the extraneous materials. Subsequently, the remaining residues were dissolved in 0.01 mL^–1^ PBS (phosphate buffer solution). The final endogenous GA concentration was estimated using a GA ELISA Kit (Qingdao Sci-tech Innovation Quality Testing Co., Ltd., Qingdao, China).

### RNA Extraction and Gene Expression Assays

Total RNA was extracted from 0.1 g of composite tomato leaves using an RNAsimple Total RNA Kit (Tiangen, Beijing, China, DP419) as per the manufacturer’s instructions. Total RNA (1 μg) was reverse transcribed to generate cDNA using the SuperScript First-strand Synthesis System (Takara, Tokyo, Japan). qRT-PCR (quantitative real-time PCR) analyses were executed employing ChamQ Universal SYBR qPCR Master Mix (Vazyme Biotech Co., Ltd., China) and the qPCR run in the StepOnePlus^TM^ Real-Time PCR System (Applied Biosystems, Foster City, CA, United States). The gene-specific primers were made based on the cDNA sequences, and the reference gene *Actin* was used (Supplementary Table S1). The relative gene expression was determined according to Shen et al. (2019).

### Statistical Analysis

The whole experiment repeated at least three independent biological replicates for the analysis of each component. Data were statistically analyzed by one-way analysis of variance (ANOVA) using SPSS 21.0 software (SPSS Inc., Chicago, IL, United States), and the significance of mean differences between treatments was analyzed with Tukey’s honestly significant difference test (HSD) at *P* < 0.05.

## Results

### Exogenous Application of Melatonin Delays Heat-Induced Leaf Senescence in Tomato Seedlings

**FIGURE 1:**
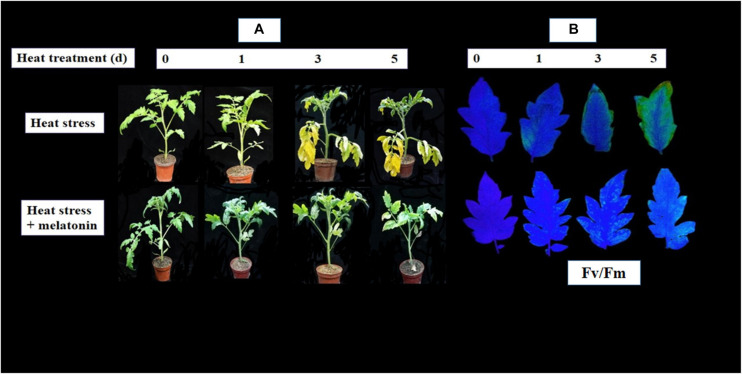
Exogenous application of melatonin (100 μM) delays heat induced (38/28°C for 5 days) leaf senescence in tomato. **(A)** Phenotypic appearance of tomato leaves during heat stress in presence or absence of melatonin treatment and **(B)** Chlorophyll fluorescence imaging with Fv/Fm of tomato leaves during heat stress in presence or absence of melatonin treatment.

**FIGURE 2:**
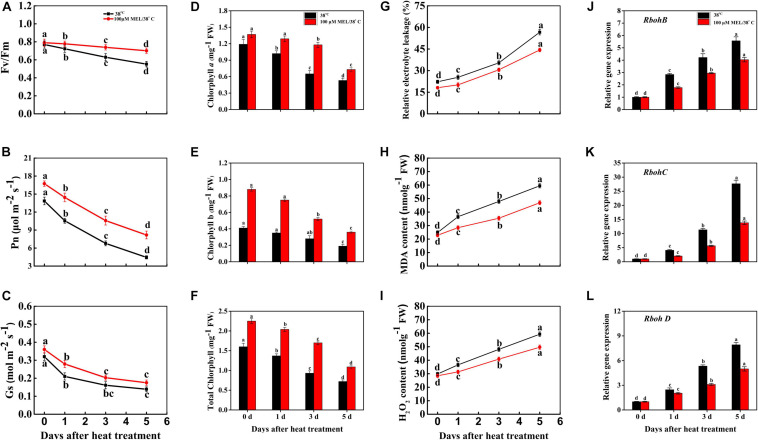
Effects of exogenous melatonin treatment (100 μM) on senescence associated physiological attributes in tomato under heat stress (38/28°C for 5 days). **(A)** Changes of Fv/Fm value, **(B)** net photosynthetic rate (P*n*), **(C)** stomatal conductance (G*s*), **(D–F)** changes of Chlorophyll content, **(G)** relative electrolyte leakage, **(H)** malondialdehyde (MDA), **(I)** hydrogen per oxide (H_2_O_2_) content, **(J–L)** relative expression of Rbohs genes during heat stress with or without of melatonin treatment. Different letters denote the significant variations between the treatments and the average values were measured by Tukey’s Honestly Significant Difference (HSD) test at *P* < 0.05. Data represented as the mean ± standard error of triplicate biological replicates.

As presented in Figure 1A, after 3 d of heat stress, the seedling leaves started to become yellow, which was prominent at day 5. However, MT-treated tomato leaves still had greener leaves than the heat-stressed seedlings on both days (Figure 1A). An analogous pattern was observed for non-invasive chlorophyll fluorescence (Figure 1B). As expected, senescence-related physiological attributes, in particular, the maximum PSII quantum yield (Fv/Fm) ratio and the total chlorophyll pigment content declined significantly following stress progression, and their values were significantly elevated in MT-treated leaves, which were approximately 1.26- and 1.51-fold those of the heat-stressed leaves, respectively, at 5 d of heat stress treatment (Figures 2A,D,E). However, the maximum chlorophyll contents were preserved in the MT-treated plants compared with melatonin-free plants. In response to heat stress, the net photosynthetic rate (P*n*) and stomatal conductance (G*s*) decreased throughout the experimental period; the rate of decline was more pronounced in non-treated seedlings than in melatonin-treated plants (Figures 2B,C).

### Melatonin Reduces Oxidative Damage and Modulates the Expression of the Rbohs Gene Under Heat Stress

We monitored relative electrolyte leakage (REL), malondialdehyde (MDA), and H_2_O_2_ contents to investigate the oxidative damage of heat-stressed seedlings. As shown in Figure 2, along with the progression of stress duration, the content of the abovementioned stress markers obviously increased in heat-stressed seedlings, while prior spraying of 100 μM MT profoundly alleviated these stress markers (REL, MDA, and H_2_O_2_ decreased in MT-treated leaves by 27.72, 26.78, and 19.48%, respectively, relative to their melatonin-free counterparts at 5 d of heat treatment), indicating that MT-treated leaves accumulated lower amounts of ROS (Figures 2G–I). Genes encoding the ROS-forming enzyme RBOH have been widely documented to be induced under stress conditions, and the relative expression of *RbohB*, *RbohC*, and *RbohD*-like was markedly elevated throughout the stress duration (Figure 2), reaching approximately 5. 57-, 27. 67-, and 7.92-fold from the initial time to 5 d of stress, respectively. In contrast, MT-treated seedlings showed downregulation of the expression of the same genes compared to heat-stressed seedlings, accounting for 1. 37-, 1. 58-, and 2.00-fold lower expression at 5 days of stress, respectively (Figures 2J–L).

### Melatonin Treatment Inhibited the Expression of Chlorophyll Degradation and Senescence Marker Genes During Heat Stress

**FIGURE 3:**
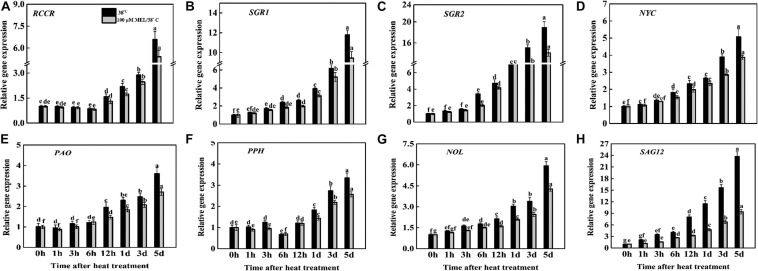
Effects of exogenous melatonin treatment on the transcript abundance of **(A–G)** chlorophyll catabolic genes (*RCCR, SGR1*, *SGR2*, *NYC*, *PAO*, *PPH*, and *NOL*) and **(H)** senescence associated gene (*SAG12*) during heat stress with or without of melatonin treatment. Different letters denote the significant variations between the treatments and the average values were measured by Tukey’s Honestly Significant Difference (HSD) test at *P* < 0.05. Data represented as the mean ± standard error of triplicate biological replicates.

Leaf yellowing is the most apparent sign of senescence, resulting in degradation of leaf chlorophyll mediated by chlorophyll catabolic genes (CCGs). The transcript abundance of chlorophyll degradation-related genes (*SGR1*, *SGR2*, *NYC*, *NOL*, *PPH*, *PAO*, and *RCCR*) and senescence marker genes (*SAG12*) were checked as seedlings sprayed with melatonin or without melatonin under heat stress. The transcript abundance of all CCGs and senescence marker genes was significantly upregulated throughout the treatment period (Figure 3). Compared to all other CCGs, the highest transcript abundance was observed for the *SGR1* and *SGR2* genes, as evidenced by 11.79- and 18.82-fold higher transcripts at 5 d of heat treatment relative to the early stage of treatment (0 h). Conversely, pretreatment with MT significantly repressed the expression of those genes at 5 d in comparison with melatonin-free heat-treated plants, as evidence by 22.03% lower *RCCR*, 24.76% reduction in *SGR1*, 30.78% reduction in *SGR2*, 31.00% decline in *NYC*, 33.21% decline in *PAO*, 30.35% reduction in *PPH*, and 38.31% lower *NOL* (Figure 3). The expression pattern of the senescence marker gene (*SAG12*) showed the same trend as the chlorophyll degradation genes. The transcript abundance of *SAG12* was upregulated with the progression of treatment duration both in MT-treated and MT-free plants, but its expression was remarkably lower (2.51-fold from only heat-stressed plants at day 5) in MT-treated tomato leaves from the initial treatment to the end of the experiment (Figure 3H).

### Exogenous Melatonin Application Induces Endogenous Melatonin and Upregulates Melatonin Synthesis Genes Under Heat Stress

**FIGURE 4:**
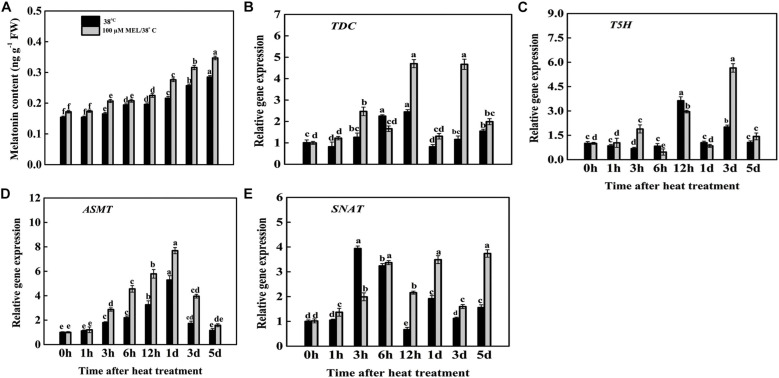
Effects of exogenous melatonin treatment on the **(A)** endogenous melatonin content and **(B–E)** transcript abundance of melatonin biosynthesis genes (*TDC, T5S, ASMT*, and *SNAT*) during heat stress with or without of melatonin treatment. Different letters denote the significant variations between the treatments and the average values were measured by Tukey’s Honestly Significant Difference (HSD) test at *P* < 0.05. Data represented as the mean ± standard error of triplicate biological replicates.

Endogenous melatonin content was measured at 0, 1, 3, 6, 12 h, 1, 3, and 5 days after heat stress in both melatonin-treated and melatonin-free seedlings (Figure 4). Melatonin content was elevated under heat stress, and with the progression of treatment duration, its content was increased, and the highest melatonin content was recorded at 5 d of heat treatment, at 1.85-fold higher than the initial time (0 h) of treatment. In contrast, melatonin addition further led to marked elevation in endogenous melatonin content from the beginning to the last day of stress. The endogenous melatonin content in MT-treated heat-stressed seedlings at 5 d reached 0.347 ng g^–1^ FW, which was 2.01-fold higher than that at the initial time of treatment (0 h) and 1.21-fold greater than that in seedlings subjected to only heat stress at 5 d of treatment (Figure 4A).

Melatonin-treated heat-stressed tomato seedlings significantly upregulated the transcript abundance of melatonin synthesis genes, namely, *TDC*, *T5H*, *SNAT*, and *ASMT* (Figures 4B–E). The transcript level of *TDC* gradually increased after 1 h of treatment, reached a peak at 12 h, and then decreased its expression. *TDC* expression again peaked at 12 h in tissues that received melatonin, and it was 4.67-fold higher than that in the early stage of treatment (0 h). Conversely, only heat-treated seedlings suppressed *TDC* expression from the early stage to the end of treatment and showed higher expression after 12 h of heat stress. *TDC* expression in melatonin-treated seedlings was 90.61% higher than that in only heat-stressed seedlings at 12 h of treatment (Figure 4B). The transcript abundance of *T5H* in both heat-stressed seedlings with or without melatonin treatment fluctuated, and obviously, the expression was higher in pretreated melatonin-stressed tissues. The transcript level of *T5H* in melatonin-treated seedlings reached a peak at 3 d after treatment, and it was 5.65-fold higher than that in the initial stage of treatment, while the expression in melatonin-free treated seedlings was 181.09% lower than that in melatonin-treated plants at 3 d of heat stress (Figure 4C). The transcript levels of *ASMT* and *SNAT* in the untreated tomato plants were lower from the initial stage to the final stage of stress. *ASMT* and *SNAT* expression peaked at 1 and 5 days of treatment and was 7.69- and 3.74-fold higher, respectively, than expression at the initial time (0 h) (Figures 4D,E). In summary, the imposition of high temperature repressed melatonin biosynthesis genes due to the inhibition of melatonin production during the stress period. As expected, melatonin pretreatment enhanced these aforementioned synthesis genes more intensely throughout the treatment period.

### Effects of Melatonin on Endogenous ABA Content and Its Biosynthesis Pathways

**FIGURE 5:**
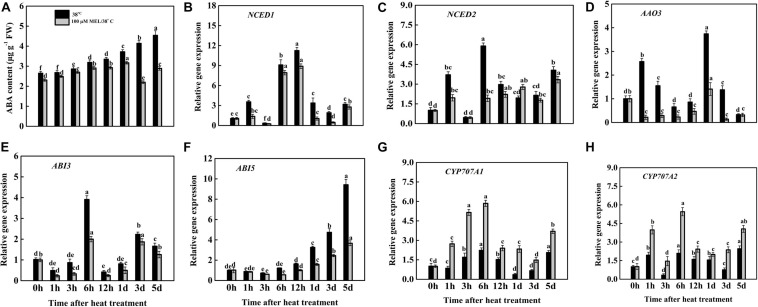
Effects of exogenous melatonin treatment on the **(A)** endogenous ABA content and **(B–D)** transcript abundance of ABA biosynthesis genes (*NCED1, NCED2*, and *AAO3*), **(E,F)** ABA signaling genes (*ABI3* and *ABI5*), **(G,H)** ABA of catabolic genes (*CYP707A1*, and *CYP707A2*) during heat stress with or without of melatonin treatment. Different letters denote the significant variations between the treatments and the average values were measured by Tukey’s Honestly Significant Difference (HSD) test at *P* < 0.05. Data represented as the mean ± standard error of triplicate biological replicates.

ABA is an effective modulator that accelerates leaf senescence (Liang et al., 2014; Mao et al., 2017). To assess whether the addition of melatonin modifies the endogenous production of ABA, the ABA concentration in tomato leaves under stressed conditions was determined. We observed a substantial increase in ABA volume both in the presence and absence of melatonin-treated plants with the progression of the heat stress period, but the ABA concentration declined in melatonin-treated plants. The lowest amount of accumulation was seen on day 3 and was approximately 51.69% lower in tissues treated with melatonin than in tissues treated with heat stress alone (Figure 5A). The highest melatonin accumulation was found in only heat-stressed seedlings at day 5, and it was recorded as 36.34% higher than that in seedlings that received melatonin. In contrast, the endogenous ABA content was markedly reduced in the ST treatment but was higher than that in the control plants. Melatonin plus ST treatment further decreased ABA accumulation under heat stress, implying that melatonin controls ABA production under heat stress (Figure 7A). In addition to justifying whether the repression of senescence by melatonin has been correlated with the modulation of ABA biosynthesis or signaling, the relative transcripts of the core genes associated with ABA biosynthesis and signaling have been investigated (Figure 5). The mRNA levels of ABA biosynthetic genes *NCED1*, *NCED2*, and *AAO3* were upregulated in heat-stressed leaves to varying degrees throughout the treatment duration. The transcript levels of *NCED1*, *NCED2*, and *AAO3* in heat-treated seedlings peaked at 12, 6 h, and 1 days after stress treatment and were 11. 25-, 5. 9-, and 3.74-fold higher than those in the initial stage of treatment (0 h), respectively (Figures 5B–D), and the expression of these genes was inhibited by melatonin treatment. In the ABA signaling pathway, *ABI3* and *ABI5* encode essential transcription factors, which increased their transcript abundance under high-temperature conditions to varying magnitudes, but the expression of these two genes was repressed in MT-treated plants throughout the stress period, and *ABI3* and *ABI5* decreased 37.74 and 61.14%, respectively, at 5 d of treatment compared to the heat-stressed seedlings (Figures 5E,F). In addition, to further confirm the contribution of melatonin to ABA modification under high-temperature conditions, we also quantified the mRNA levels of two ABA catabolism genes, *CYP707A1* and *CYP707A2*. As expected, the relative transcripts of these two genes were significantly upregulated to varying extents in melatonin-treated tissues compared with heat-stressed seedlings. The highest expression of *CYP707A1* and *CYP707A2* was observed in melatonin-pretreated tissues 6 h after treatment and increased 162.33 and 160.76%, respectively, relative to melatonin-free heat-stressed seedlings (Figures 5G,H). Altogether, the above findings imply that melatonin addition delays heat-induced leaf senescence in tomato, and it might be closely associated with reduced endogenous ABA production along with regulation of the ABA metabolic pathway.

### Effects of Exogenous Melatonin on Endogenous GA Accumulation and Its Biosynthesis Pathways

**FIGURE 6:**
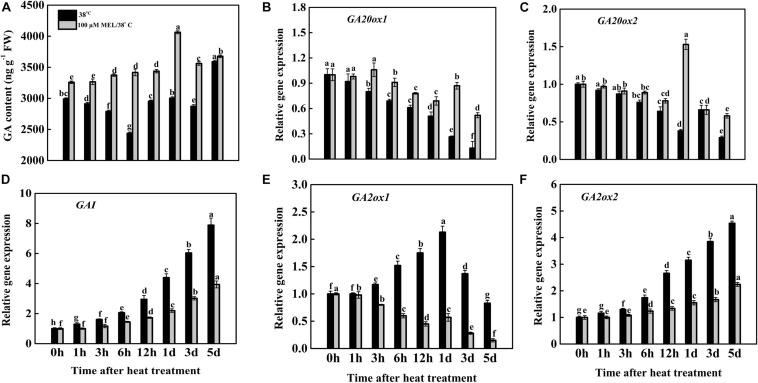
Effects of exogenous melatonin treatment on the **(A)** endogenous GA content and **(B,C)** transcript abundance of GA biosynthesis genes (*GA20ox1* and *GA20ox2*), **(D)** GA signaling genes (*GAI*), **(E,F)** GA of catabolic genes (*GA2ox1* and *GA2ox2*) during heat stress with or without of melatonin treatment. Different letters denote the significant variations between the treatments and the average values were measured by Tukey’s Honestly Significant Difference (HSD) test at *P* < 0.05. Data represented as the mean ± standard error of triplicate biological replicates.

**FIGURE 7:**
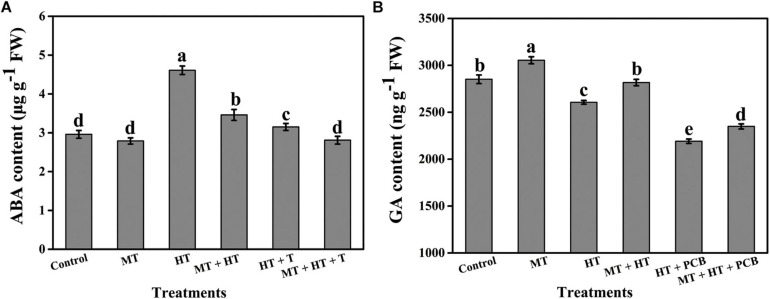
Effects of 1 mM sodium tungstate (ST, an ABA biosynthesis inhibitor) and 1 mM paclobutrazol (PCB, a GA biosynthesis inhibitor) on the **(A)** endogenous ABA and **(B)** endogenous GA content during heat stress with or without of melatonin treatment. Different letters denote the significant variations between the treatments and the average values were measured by Tukey’s Honestly Significant Difference (HSD) test at *P* < 0.05. Data represented as the mean ± standard error of triplicate biological replicates.

To determine the interaction of GA mediating MT-induced heat tolerance, we estimated the endogenous GA accumulation of tomato plants. However, heat stress resulted in decreases in the GA content throughout the treatment period to varying degrees. Exogenous melatonin pretreatment significantly elevated the GA content under heat stress. The maximum GA accumulation was found 1 d after heat stress, and it was 1.24-fold higher than the initial time (0 h) of heat treatment and 1.35-fold higher on the same day compared to heat-stressed leaves (Figure 6A). The endogenous GA content profoundly decreased in the PCB treatment under heat stress and was lower than that in control, while treatment with melatonin plus PCB under heat stress slightly increased the GA content, suggesting that MT-mediated heat tolerance is associated with GA (Figure 7B). In addition to verifying whether the inhibition of senescence by MT has been associated with the regulation of GA biosynthesis pathways, the mRNA levels of the core genes associated with GA synthesis, signaling, and catabolism were investigated (Figure 6). We assumed that the relative expression of GA biosynthesis genes (*GA20ox1* and *GA20ox2*) might be modulated by melatonin treatment under heat stress. As displayed in Figure 6, mRNA level analysis revealed that the transcript abundances of *GA20ox1* and *GA20ox2* were downregulated under only heat stress conditions, while the addition of melatonin throughout the heat treatment duration reversed the downregulation of the expression of these genes. The transcription levels of *GA20ox1* and *GA20ox2* in melatonin-treated plants reached their peaks after treatment for 3 h and 1 d; their expression was 1.35- and 4.02-fold higher, respectively, than heat-stressed tissues at the same time points (Figures 6B,C). DELLA proteins (GAI and RGA) coordinate with key regulatory elements, modifying downstream genes transcriptionally to suppress plant growth, whereas GA enhances plant growth and development by suppressing DELLA inhibition to stimulate GA (Davière and Achard, 2013). To obtain more insight into how GA and melatonin interact to mitigate heat-induced leaf senescence, we also quantified the GA signaling repressor gene *GAI*. As shown in Figure 6D, with the progression of heat stress duration, the abundance of *GAI* transcripts was significantly upregulated until the end of the experiment; however, exogenous application of melatonin constantly suppressed this gene expression. On day 5, the transcript level of *GAI* in melatonin-treated tissue was 50.06% lower than that in only heat-stressed seedlings (Figure 6D), indicating that melatonin might play a key role in inhibiting DELLA production by suppressing *GAI* expression, which helps to delay heat-induced leaf senescence. The key synthesized catabolic bioactive GA enzyme is GA2ox, which encodes for the negative regulation of GA metabolism. We also checked the expression of two crucial GA2ox-encoding genes (*GA2ox1* and *GA2ox2*); our qRT-PCR results showed that the overall transcription levels of these two genes were upregulated under heat-stressed tomato leaves; however, the expression of these genes was significantly suppressed by melatonin treatment under high temperature (Figures 6E,F). Accordingly, our results indicate that heat-induced leaf senescence is suppressed in tomato seedlings with melatonin treatment, which may be strongly interlinked with endogenous GA content as well as GA biosynthesis and signaling pathways.

### Pearson’s Correlation Coefficient Relationship Among the Key Genes of Melatonin, GA and ABA Pathways

To gain more understanding among the relationship of the three key molecules MT, GA and ABA biosynthesis, we performed Pearson’s coefficient test (Supplementary Tables S2, S3). The correlation has differentially shown among these three biosynthesis pathways at different treatment conditions. Melatonin biosynthesis gene was significantly positive correlation with the GA biosynthesis gene, whereas, it strongly negative relation with ABA signaling pathway related gene. Similarly, GA biosynthesis was significantly opposite to ABA biosynthesis; by contrast, the ABA signaling pathway related gene was positively correlated with the GA signaling gene. In summary, we concluded that melatonin has a positive relation with GA to mitigate heat-induced leaf senescence and vice versa with ABA at the various treatment combinations.

## Discussion

A set of phytohormones stimulates stress-induced or natural leaf senescence, whether abscisic acid, ethylene, jasmonic acid promoting senescence, and auxin, cytokinin (CK), and gibberellins prolonging the senescence process (Fan Z.-Q. et al., 2018; Tan et al., 2018). Melatonin acts as an anti-senescence factor, suppresses chlorophyll catabolism and other senescence-associated promotion of gene expression (Shi et al., 2015a; Zhang et al., 2016a; Arnao and Hernández-Ruiz, 2019). In the current experiment, we found that melatonin application significantly suppressed chlorophyll degradation-associated gene (*SGR1*, *SGR2*, *NYC*, *NOL*, *PPH*, *PAO*, and *RCCR*) and senescence marker gene (*SAG12*) expression (Figure 3). The total chlorophyll content, Fv/Fm ratio, and photosynthetic attributes (P*n* and G*s*) declined in only heat-stressed tomato seedlings (Figures 2A–F). Conversely, melatonin pretreatment effectively elevated these processes, leading to a substantial decline in pigment loss and maintaining photosystem integrity, implying that melatonin played a vital role in mitigating heat-induced leaf senescence in tomato (Figure 2). In agreement with our results, slowed leaf senescence has been documented in melatonin treated Chinese flowering cabbage (Tan et al., 2020), rice (Liang et al., 2015), bentgrass (Ma et al., 2018a), and kiwifruit (Liang et al., 2018). Plants face oxidative stress due to over ROS production, which directly participates in senescence acceleration (Choudhary et al., 2020). A constant elevation of REL, MDA, and H_2_O_2_ indicated a decline in cell membrane integrity through excess ROS production, and melatonin addition significantly attenuated this oxidative damage (Figures 2G–I). RBOHs are widely studied enzymatic stocks of ROS generation and play critical roles in altering ROS production (Wang et al., 2019). The transcript levels of RBOH (*RbohB*, *RbohC*, and *RbohD*) increased continuously with increasing treatment period, while exogenous spraying of melatonin differentially repressed *RbohB*, *RbohC*, and *RbohD* expression, resulting in lowered accumulation of ROS (Figures 2J–L). These results suggest that the lowered generation of ROS in melatonin pretreated seedlings is indirectly related to inhibition of RBOH gene expression. Our results are also in line with recent findings and indicate that the protective effect of melatonin on stress-induced ROS accumulation is related to *RBOH* gene regulation as well as other metabolite functions (Jahan et al., 2019a; Tan et al., 2020).

Melatonin pretreatment or overexpression or transient expression of melatonin biosynthesis genes might enhance the *in vivo* melatonin level and increase plant stress tolerance (Ahammed et al., 2019). In the current experiment, exogenous addition of melatonin led to elevated melatonin content, and the transcript abundances of *TDC*, *T5S*, *ASMT* and *SNAT* were significantly downregulated in tissues subjected to only heat stress (Figure 4). Correspondingly, the decline in mRNA levels of these biosynthetic genes was suppressed in melatonin-treated tissues exposed to high temperature. A couple of former studies indicated that melatonin content, and melatonin biosynthesis genes expression were significantly upregulated upon melatonin treatment (Zhang et al., 2017; Ma et al., 2018a; Tan et al., 2020).

Melatonin could potentially interact with plant hormones or signaling molecules, employing beneficial roles in stress management. Several recent studies have indicated that melatonin is symbiotic or contrary to other phytohormones throughout physiological processes in stress responses (Arnao and Hernández-Ruiz, 2014, 2015, 2019; Reiter et al., 2015; Kanwar et al., 2018). The elevated levels of ABA promote leaf senescence (Yang et al., 2002). Melatonin treatment significantly suppressed ABA accumulation as well as ABA biosynthesis genes expression and upregulated the expression of ABA catabolic genes under salt and water stress (Zhang H. J. et al., 2014; Li et al., 2015). In this study, heat-stressed induced higher ABA levels and elevated expression of ABA biosynthesis (*NCED1*, *NCED2*, and *AAO3*) and signaling transcription factor (*ABI3* and *ABI5*) genes, while ABA catabolic genes (*CYP707A1* and *CYP707A2*) were suppressed under the same treatment (Figure 5). However, the opposite trends were observed in the melatonin pretreated plants. Our findings were consistent with previous work and found that melatonin application relieved high temperature-induced leaf senescence by repressing ABA induction, lowering the expression of ABA synthesis and signaling genes (Zhang et al., 2016a) and upregulating catabolic gene expression (Li et al., 2015). In addition, ABA biosynthesis and signaling transcription factors are elevated during stress as well as in natural senescence environments (Finkelstein and Rock, 2002). In line with these findings, it can be hypothesized that the addition of melatonin decreases ABA production and enhances melatonin contents by concurrently inhibiting ABA biosynthetic gene expression and increasing melatonin biosynthesis gene expression, thus ultimately inhibiting heat-induced leaf senescence damage.

Bioactive GA is an essential element that plays an active role in delaying leaf senescence in plants subjected to stressful environments (Fan J. et al., 2018; Xiao et al., 2019). In the present investigation, the content of endogenous GA accumulation along with GA biosynthetic encoding enzyme gene (*GA20ox1* and *GA20ox2*) expression was significantly elevated in melatonin-treated plants under heat stress conditions, and the opposite trend was notified in only heat-stressed seedlings (Figure 6). Furthermore, melatonin application significantly repressed the transcript level of the DELLA protein-encoding gene *GAI* (used for GA deactivation or as a GA suppressor) as well as GA catabolic regulating gene (*GA2ox1* and *GA2ox2*) expression (Figure 6D). Our findings are also supported by previous works, which noted that the application of GA delays natural or stress-induced leaf senescence (Fan J. et al., 2018; Xiao et al., 2019). The supplementation with melatonin increases active GAs under salinity stress in cucumber seedlings by amplifying GA biosynthetic genes (Zhang N. et al., 2014) and delays plant senescence by preventing ROS production and optimizing antioxidant enzyme activities (Wang et al., 2017). Collectively, the cumulative effects of elevated melatonin content, melatonin biosynthesis and GA signaling gene transcription levels could result in increased heat tolerance and delayed leaf senescence in tomato.

Generally, the plant hormones GA and ABA interact antagonistically at different plant growth stages as well as under diverse stress conditions, including high temperature (Ahammed et al., 2020b). The cross-talk between the GA and ABA signaling pathways and the inconsistent combination between these two plant growth regulators also directly activate corresponding stress responses (Liu and Hou, 2018; Ahammed et al., 2020b). High temperature enhances the higher accumulation of ABA and suppresses GA content in Arabidopsis while elevating ABA levels triggers upregulation of ABA biosynthesis enzyme genes, but lower GA accumulation occurs due to downregulation of GA biosynthesis enzyme genes under the same stress conditions (Toh et al., 2008). DELLAs govern a group of downstream genes at the transcript level by emulating TFs implicated in ABA signaling. For example, the GA signaling suppressors GAI and RGA in Arabidopsis act upon temperature stress by correlating to ABA signaling transcription factors, including *ABI3* and *ABI5*, and explicitly initiating small ubiquitin-related modifier (SOM), which revamps ABA and GA biosynthesis in Arabidopsis (Lim et al., 2013). We observed that melatonin application significantly influenced both GA and ABA biosynthesis and signaling pathways in the current investigation. In conclusion, the above results indicated that there might be a strong cross-connection among these three signaling molecules and that melatonin treatment repressed heat-induced leaf senescence in tomato either directly or indirectly by redesigning GA and ABA metabolism or modulating chlorophyll catabolic pathways.

**FIGURE 8:**
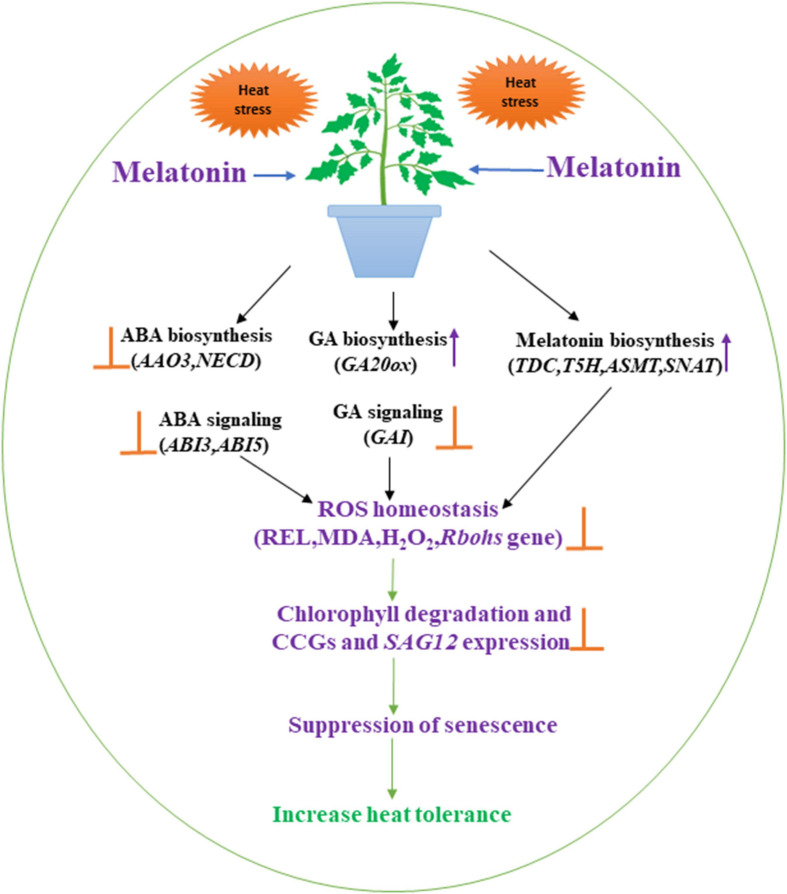
A probable mechanism of melatonin-mediated heat-induced leaf senescence resulting in coordination with ABA and GA biosynthesis and signaling pathway. The arrow denotes increases and bar denotes decreases.

Finally, as depicted in Figure 8, the present study revealed that melatonin application prolongs heat-induced leaf senescence of tomato seedlings through the upregulation of *in vivo* melatonin and GA content, whereas, inhibition of ABA formation and reduction of chlorophyll degradation. The positive contributions of melatonin and GA in enhancing heat tolerance were indicated by plant physiological attributes and suppression of ROS overproduction. These findings reveal the cross-talk among the three molecules and related directly or partially to their biosynthesis pathways. Thus allowing mitigation of the heat tolerance of tomato plants and facilitating an understanding of their interactions. Further studies are required via transgenic or VIGS approaches to insight more understanding about their interactions.

## Data Availability Statement

The original contributions presented in the study are included in the article/Supplementary Material, further inquiries can be directed to the corresponding author/s.

## Author Contributions

SG contributed to conceptualization, design of the experiment, methodology, and fund acquisition. MJ performed the experiment and contributed to data curation and original draft preparation. YW revised the manuscript and contributed to software. SS contributed to supervision and editing. MH and DH prepared the figure. AE-Y revised the manuscript. NA contributed to data analysis. MA contributed to the collection of literature. JS supervised and reviewed the original manuscript. All authors contributed to the article and approved the submitted version.

## Conflict of Interest

The authors declare that the research was conducted in the absence of any commercial or financial relationships that could be construed as a potential conflict of interest.

## Supplementary Material

Supplementary Table 1 List of primer pairs used in this experiment.

Supplementary Table 2 Pearson’s correlation coefficient relationship among the key genes of melatonin, GA and ABA pathways (For only heat stress).

Supplementary Table 3 Pearson’s correlation coefficient relationship among the key genes of melatonin, GA and ABA pathways (For Melatonin and heat stress).
