# Supplementary material for: Melatonin Pretreatment Confers Heat Tolerance and Repression of Heat-Induced Senescence in Tomato Through the Modulation of ABA- and GA-Mediated Pathways
Source: Front Plant Sci. 2021 Mar 25;12:650955. doi: 10.3389/fpls.2021.650955 (PMC8027311; doi:10.3389/fpls.2021.650955)
Supplement: Supplementary Table 1 — List of primer pairs used in this experiment. [file Data_Sheet_1.docx]

| Gene Name | Forward primer (5ˊ to 3 ˊ) | Reverse primer (5ˊ to 3 ˊ) |
| --- | --- | --- |
| *TDC* | TGCTCTAGACTCCGTCACCACAAAGC | CGCGGATCCCCCAGAAATCCAGCCAC |
| *T5S* | TGCTCTAGATGATTTCGTCTCGTTCT | CGCGGATCCCTCTTCTTCATCCCACT |
| *ASMT* | TGCTCTAGAGCTTTCTTGTTCGCTAT | CGCGGATCCGTCCATCAGGGAGTGTC |
| *SNAT* | TGCTCTAGATCTTCCCTACCCACTAT | CGCGGATCCACATCAACATCTCCTCC |
| *NCED1* | TTTTACGCTCGTGGGCTCTT | GCCGGTGGGTGTTACCTTTA |
| *NCED2* | GCGAGGCTATTGTTGTTCT | CAATCCAGCATTAGCAACTC |
| *AAO3* | AGTTTAGTGCAAGGCGGGTT | TCTATCTCCACCTCGCTGACA |
| *CYP707A1* | AGAGAGGCTGTAGCTGAGTGG | TTGGCAAGTTCATTCCCTGGAC |
| *CYP707A2* | GCAATGAAAGCGAGGAAAGAGC | TCGAGCTGCAAAGATGACTCC |
| *ABI3* | TGTGTTACCAAAGAAAGAAGCAG | GGCTGCATGCCATCTGATTT |
| *ABI5* | ATCCGCCACTTCCACAAACC | AACACCGCCTGTGACTCCA |
| *SGR1* | TTCTCAGTTGCAAGGTTGGT | CCTTGAGAACCACAGGGAGT |
| *SGR2* | CCTTGGAGCACACACGAAC | TTCCACAGGTTCACAAGACGA |
| *PAO* | AAATGCCCTCATCGTCTTGC | AGCACAAGCTCTTGGAGACT |
| *PPH* | GGAGGGAGCAAGTACGCTAT | AAGGCGTAGCATTCAGCAAG |
| *NYC* | CAGGCAGCTTCAATCATCCC | TAGGTCGGTTAGGACCATGC |
| *NOL* | CTCAGATGGAAGACCAACCCC | TTGTGACCATTCCAGGCGA |
| *RCCR* | TTCCTCTTCTCAGGTTGATTTCAT | CCTTTCGCGGAGGTAGATCA |
| *SAG12* | GTGGCTAATCAACCGGTGTC | CACTTGCAGCACCATATCCC |
| *RBOHB* | CGTGAAGACTCTGTGGCTGT | GCACCGTTTGATTTGGTGCT |
| *RBOHC* | GCGGTGGGAGTTTTTGATGC | TGTTTTGATTGAGAAAGTTGAACCA |
| *RBOHD* | GGGTCATTGCGTTTGTGTGG | AGCCCTGTGGCTTTGACATT |
| *GA20ox1* | TTCTTTCTGGTGACCCCGAC | AACGCCATTCATCGTCCACA |
| *GA20ox2* | GGGGCATTCCTTTCTGGTGA | GTGGTCCAGTTCCTAACGCA |
| *GAI* | GCGATGGTTACAGGGTGGAA | ATAGCTTCCAGGCGGAGGTA |
| *GA2ox1* | CACCTCACCCGAATACTGCT | ACGAGTGTCCCCAAGTCTCA |
| *GA2ox2* | ACCCGACTCCAAGAACCTCA | TGTTCGACCCGACCACAATC |
| *Actin* | TGGTCGGAATGGGACAGAAG | CTCAGTCAGGAGAACAGGGT |

Table S1 The primer pairs used in this experiment

**Table S2** **Pearson’s correlation coefficient relationship among the key genes of melatonin, GA and ABA pathways (For only heat stress)**

| **. Correlation is significant at the 0.01 level (2-tailed). |
| --- |
| *. Correlation is significant at the 0.05 level (2-tailed). |

|  | | | | | | | | | | | | | | | |
| --- | --- | --- | --- | --- | --- | --- | --- | --- | --- | --- | --- | --- | --- | --- | --- |
|  | ***NCED1*** | ***NCED2*** | ***ABI5*** | ***ABI3*** | ***CYP707A1*** | ***CYP707A2*** | ***SNAT*** | ***T5H*** | ***TDC*** | ***ASMT*** | ***GA20ox1*** | ***GA20ox2*** | ***GAI*** | ***GA2ox1*** | ***GA2ox2*** |
| ***NCED1*** | **1** |  |  |  |  |  |  |  |  |  |  |  |  |  |  |
| ***NCED2*** | **.736^**^** | **1** |  |  |  |  |  |  |  |  |  |  |  |  |  |
| ***ABI5*** | **-.160** | **.196** | **1** |  |  |  |  |  |  |  |  |  |  |  |  |
| ***ABI3*** | **.463^*^** | **.610^**^** | **.143** | **1** |  |  |  |  |  |  |  |  |  |  |  |
| ***CYP707A1*** | **.447^*^** | **.452^*^** | **.157** | **.486^*^** | **1** |  |  |  |  |  |  |  |  |  |  |
| ***CYP707A2*** | **.560^**^** | **.766^**^** | **.415^*^** | **.215** | **.367** | **1** |  |  |  |  |  |  |  |  |  |
| ***SNAT*** | **.400** | **.499^*^** | **.521^**^** | **.334** | **.332** | **.539^**^** | **1** |  |  |  |  |  |  |  |  |
| ***T5H*** | **-.183** | **-.226** | **.220** | **.035** | **-.254** | **-.428^*^** | **-.328** | **1** |  |  |  |  |  |  |  |
| ***TDC*** | **.171** | **-.096** | **.135** | **.014** | **.024** | **-.273** | **-.152** | **.854^**^** | **1** |  |  |  |  |  |  |
| ***ASMT*** | **.423^*^** | **.021** | **-.081** | **-.011** | **-.239** | **-.016** | **.460^*^** | **.117** | **.305** | **1** |  |  |  |  |  |
| ***GA20ox1*** | **-.022** | **-.209** | **-.886^**^** | **-.241** | **-.091** | **-.267** | **-.581^**^** | **-.460^*^** | **-.444^*^** | **-.272** | **1** |  |  |  |  |
| ***GA20ox2*** | **-.112** | **-.242** | **-.789^**^** | **-.008** | **-.024** | **-.447^*^** | **-.785^**^** | **-.084** | **-.152** | **-.467^*^** | **.847^**^** | **1** |  |  |  |
| ***GAI*** | **-.098** | **.166** | **.954^**^** | **.161** | **.045** | **.312** | **.526^**^** | **.410^*^** | **.330** | **.146** | **-.968^**^** | **-.845^**^** | **1** |  |  |
| ***GA2ox1*** | **.412^*^** | **.002** | **-.222** | **-.061** | **-.300** | **-.054** | **.335** | **.060** | **.230** | **.968^**^** | **-.109** | **-.330** | **.000** | **1** |  |
| ***GA2ox2*** | **.002** | **.177** | **.899^**^** | **.126** | **-.006** | **.294** | **.552^**^** | **.449^*^** | **.425^*^** | **.295** | **-.975^**^** | **-.878^**^** | **.976^**^** | **.156** | **1** |

**Table S3 Pearson’s correlation coefficient relationship among the key genes of melatonin, GA and ABA pathways (For Melatonin and heat stress)**

|  | | | | | | | | | | | | | | | |
| --- | --- | --- | --- | --- | --- | --- | --- | --- | --- | --- | --- | --- | --- | --- | --- |
|  | ***NCED1*** | ***NCED2*** | ***ABI5*** | ***ABI3*** | ***CYP707A1*** | ***CYP707A2*** | ***SNAT*** | ***T5H*** | ***TDC*** | ***ASMT*** | ***GA20ox1*** | ***GA20ox2*** | ***GAI*** | ***GA2ox1*** | ***GA2ox2*** |
| ***NCED1*** | **1** |  |  |  |  |  |  |  |  |  |  |  |  |  |  |
| ***NCED2*** | **.222** | **1** |  |  |  |  |  |  |  |  |  |  |  |  |  |
| ***ABI5*** | **-.235** | **.608^**^** | **1** |  |  |  |  |  |  |  |  |  |  |  |  |
| ***ABI3*** | **.096** | **.183** | **.347** | **1** |  |  |  |  |  |  |  |  |  |  |  |
| ***CYP707A1*** | **.334** | **-.064** | **-.234** | **.114** | **1** |  |  |  |  |  |  |  |  |  |  |
| ***CYP707A2*** | **.499^*^** | **.400** | **.111** | **.417^*^** | **.533^**^** | **1** |  |  |  |  |  |  |  |  |  |
| ***SNAT*** | **-.030** | **-.365** | **-.332** | **.129** | **.831^**^** | **.141** | **1** |  |  |  |  |  |  |  |  |
| ***T5H*** | **.545^**^** | **.188** | **.060** | **-.160** | **-.378** | **-.156** | **-.542^**^** | **1** |  |  |  |  |  |  |  |
| ***TDC*** | **.900^**^** | **.100** | **-.122** | **.178** | **.428^*^** | **.411^*^** | **.096** | **.540^**^** | **1** |  |  |  |  |  |  |
| ***ASMT*** | **.191** | **.311** | **-.117** | **-.128** | **-.045** | **-.155** | **.057** | **.260** | **-.025** | **1** |  |  |  |  |  |
| ***GA20ox1*** | **-.190** | **-.827^**^** | **-.746^**^** | **-.187** | **.082** | **-.252** | **.280** | **-.225** | **-.142** | **-.323** | **1** |  |  |  |  |
| ***GA20ox2*** | **-.233** | **-.034** | **-.387** | **-.262** | **-.135** | **-.273** | **.129** | **-.295** | **-.507^*^** | **.682^**^** | **.090** | **1** |  |  |  |
| ***GAI*** | **-.071** | **.637^**^** | **.949^**^** | **.437^*^** | **-.088** | **.190** | **-.201** | **.156** | **.059** | **.043** | **-.812^**^** | **-.390** | **1** |  |  |
| ***GA2ox1*** | **-.242** | **-.606^**^** | **-.769^**^** | **-.459^*^** | **-.047** | **-.258** | **.129** | **-.397** | **-.361** | **-.194** | **.764^**^** | **.444^*^** | **-.906^**^** | **1** |  |
| ***GA2ox2*** | **.265** | **.658^**^** | **.832^**^** | **.240** | **.013** | **.275** | **-.247** | **.377** | **.362** | **.048** | **-.855^**^** | **-.484^*^** | **.895^**^** | **-.902^**^** | **1** |

| **. Correlation is significant at the 0.01 level (2-tailed). |
| --- |
| *. Correlation is significant at the 0.05 level (2-tailed). |
